# Aging related changes of circadian rhythmicity of cytotoxic lymphocyte subpopulations

**DOI:** 10.1186/1740-3391-8-6

**Published:** 2010-05-25

**Authors:** Gianluigi Mazzoccoli, Angelo De Cata, Antonio Greco, Marcello Damato, Nunzia Marzulli, Mariangela Pia Dagostino, Stefano Carughi, Federico Perfetto, Roberto Tarquini

**Affiliations:** 1 Department of Internal Medicine, Scientific Institute and Regional General Hospital "Casa Sollievo della Sofferenza", S.Giovanni Rotondo (FG), Italy; 2 Department of Internal Medicine, University of Florence, Florence, Italy

## Abstract

**Background:**

Immunosenescence is a process that affects all cell compartments of the immune system and the contribution of the immune system to healthy aging and longevity is still an open question. Lymphocyte subpopulations present different patterns of circadian variation and in the elderly alteration of circadian rhythmicity has been evidenced. The aim of our study was to analyze the dynamics of variation of specific cytotoxic lymphocyte subsets in old aged subjects.

**Methods:**

Lymphocyte subpopulation analyses were performed and cortisol serum levels were measured on blood samples collected every four hours for 24 hours from fifteen healthy male young-middle aged subjects (age range 36-55 years) and fifteen healthy male old aged subjects (age range 67-79 years).

**Results:**

In healthy young-middle aged subjects CD20 were higher and at 06:00 h CD8+ dim correlated positively with CD16+ and positively with γδTCR+ cells, CD16 correlated positively with γδTCR+ cells At 18:00 h CD8+ dim correlated positively with CD16+ and positively with γδTCR+ cells, CD16+ correlated positively with γδTCR+ cells and a clear circadian rhythm was validated for the time-qualified changes of CD3+, CD4+, CD20+, CD25+ and HLA-DR+ cells with acrophase during the night and for the time-qualified changes of CD8+, CD8+ bright, CD8+ dim, CD16+ and γδTCR+ cells with acrophase during the day. In old aged subjects CD25, DR+ T cells and cortisol serum levels were higher, but there was no statistically significant correlation among lymphocyte subpopulations and a clear circadian rhythm was evidenced for time-qualified changes of CD3+ and CD25+ cells with acrophase during the night and for the time-qualified changes of CD8+ cells and cortisol with acrophase during the day.

**Conclusion:**

Our study has evidenced aging-related changes of correlation and circadian rhythmicity of variation of cytotoxic lymphocyte subpopulations that might play a role in the alteration of immune system function in the elderly.

## Background

There are a number of reports in the scientific literature that put in evidence a circadian rhythm of variation of total lymphocytes in the peripheral blood, of antibodies and cell mediated immune responses [1,2] and an inverse relationship with plasma cortisol concentration [3]. Alteration of circadian rhythmicity has been evidenced in the elderly. A small fraction of peripheral T cells coexpress CD4 and low levels of CD8 (CD4+CD8dim) and can have cytotoxic activity. NK receptors are constitutively expressed and inducible on CD8+ cells upon antigen exposure or the cellular stress and cell-mediated cytotoxicity functions through non-major histocompatibility complex (MHC)- or MHC-restricted mechanisms. MHC-restricted cytotoxicity is mainly mediated by CD8+ cytotoxic T lymphocytes through two distinct perforin-and Fas-based pathways resulting in tissue destruction [4]. γδ-TCR expressing T cells represent a distinct mature T-cell lineage with the capacity to proliferate in response to receptor-mediated signals and to display non-MHC-restricted cytolysis [5,6]. Natural killer (NK) cells are large granular lymphocytes that express neither αβ or γ/δ TCR nor CD3 on their surface and can lyse a number of different tumour cells. NK cells originate from bone marrow, but can mature in a variety of primary and secondary lymphoid tissues and the interaction with dendritic cells seems to be required for their optimal activation. The two key effector functions of human NK cells are killing and cytokine production and NK cells could mediate tissue damage and regulate autoimmune T-cell responses through cytokine secretion and cytotoxicity in secondary lymphoid organs [7].

Cytotoxic T lymphocytes are part of the adaptive immune system, natural killer cells are part of the innate immune system, and γδ-TCR expressing T cells may represent a functional and/or temporal bridge between this two cellular arms and may link the two major functional modality of immune response. These three cellular subsets differ in killing repertoire, but their function is of outmost importance for the body defence against foreign cells, cancer cells and cells infected with a virus.

In this study we investigated physiological variations of specific cytotoxic T lymphocyte subsets in old aged subjects.

## Methods

Subjects gave written informed consent and the study was approved by the local Scientific and Ethical Committee. Peripheral blood samples were collected at intervals of four hours for twenty four hours from fifteen healthy male young and middle aged subjects (range 36-55 years, mean age ± s.e. 44.1 ± 1.7) and fifteen healthy male old aged subjects (range 67-79 years, mean age ± s.e. 68.5 ± 1.2). Inclusion criteria were age (< 65 years for young and middle aged subjects and ≥ 65 and < 80 years for old aged subjects), BMI (> 25 and < 30), no smoking status, normal physical activity level, no psychiatric disorder, no alcohol intake, no chronic conditions, and normal blood pressure level. In all subjects healthy status was assessed by medical history and physical examination, basal screening blood and urine test, ECG, chest X ray, and upper and lower abdominal ultrasound scan. All subjects were studied in our department and were submitted to the same social routine (light/dark cycle and mealtimes). Sleep was allowed between 23:00 h (lights off) and 07:00 h (lights on). During daytime (between 07:15 h and 20:15 h), subjects stayed in the department, and standardized meals were provided at appropriate times for breakfast (07:30 h), lunch (12:30 h), and dinner (18:30 h). In each blood sample we analyzed lymphocyte subpopulations (CD3, CD4, CD8, CD16, CD20, CD25, HLA-DR, TcRδ1) on peripheral blood anticoagulated with sodium ethylenediamine tetraacetic acid (EDTA) and we measured cortisol on serum. Analyses of lymphocyte subpopulations were performed on unfixed cell preparations with a multicolor fluorescence activated cell sorter (FACScan, Becton-Dickinson FACS Systems, Sunnyvale, California) and a panel of monoclonal antibodies (mAbs) to lymphocyte surface antigens (Ortho Diagnostic Systems: OKT3, OKT4, OKT8, OK-NK, OKB20, OKT26a, OK-DR; Medical Systems: TcRδ1). Briefly, mAbs were directly conjugated with phycoerythrin (PE) and 10 μl mAbs were added to 100 ml EDTA blood in Trucount tubes (BD Biosciences, San Jose, CA). After a 15-min incubation at room temperature the erythrocites were disintegrated and after centrifugation the supernatants were washed with PBS. Non-lymphocytic cells contaminating the preparations were excluded from analysis using scatter gates set on the 90° light scatter profile. At least 10000 cells were acquired on the FACScan. Absolute counts of T cell subsets were calculated based on the proportion of the respective T cell subpopulation and on absolute counts obtained by the procedure. The number of fluorescent cells was expressed as a percentage of the total lymphocytes. To measure hormone serum concentrations blood samples were centrifuged immediately after collection and frozen at -20°C for later determination. All samples were analyzed in duplicate in a single assay; the intrassay and interassay coefficients of variation were below respectively 10% and 9% using a polarized light immuno-fluorescence assay (Cortisol TDx/TDxFLx, Abbott Laboratories, Abbott Park, Illinois, USA).

### Statistical analysis

Statistical evaluation of percentages of cells was performed by non-inferential descriptive biometric analysis (Pearson's product moment correlation coefficients and linear regression calculated for percentages of cells at each sampling time to assess temporal relationships between variations in lymphocyte subpopulations and Student's *t *test and Mann-Whitney rank sum test, as indicated, on areas under the curve, calculated according to the trapezoidal method; a *p *value ≤ 0.05 was considered significant) and by an inferential temporal descriptive biometric analysis using the methods named Single Cosinor and Population Mean Cosinor, based on a least square fit of a cosine wave to individual and group time series data, testing the occurence (whether the zero-amplitude assumption is rejected at a probability level *p *≤ 0.05) and quantifying the parameters MESOR, Amplitude and Acrophase of consistent pattern of circadian rhythm. MESOR is the acronym for Midline Estimating Statistic of Rhythm and defines the rhythm-determined average. Amplitude is the measure of one half the extent of rhythmic change in a cycle estimated by the function used to approximate the rhythm. Acrophase, measure of timing, is the phase angle of the crest time in the function appropriately approximating a rhythm, in relation to the specified reference timepoint. Rhythms with a frequency of 1 cycle per 20 ± 4 h are designated circadian, rhythms with a frequency higher than 1 cycle per 24 h are designated as ultradian, and rhythms with a frequency lower than 1 cycle per 24 h are designated as infradian [8].

## Results

**Figure 1:**
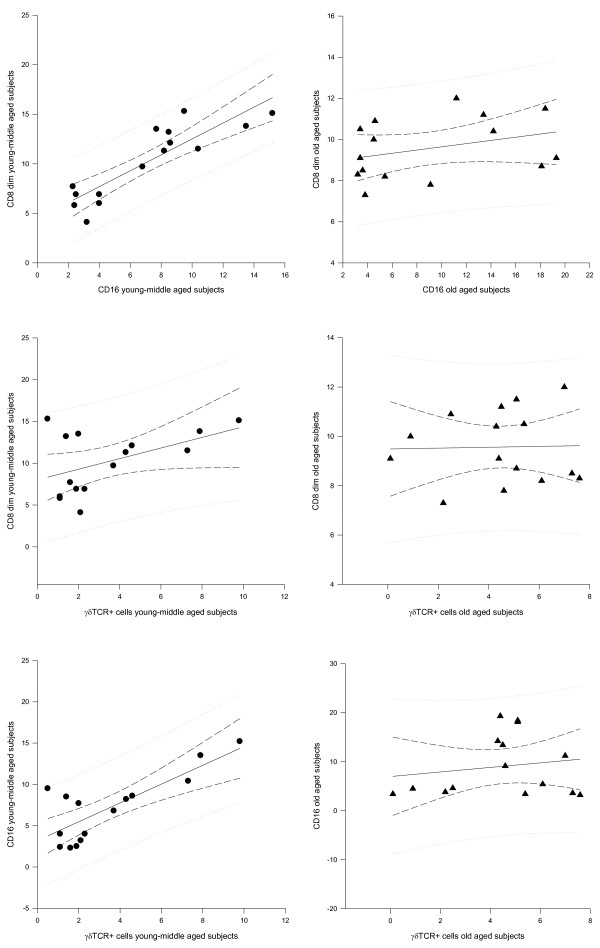
**Correlations between lymphocyte subpopulations in the photoperiod**. In the photoperiod (06:00h-10:00h-14:00h) CD8+ dim correlates positively with CD16+ *(r = 0.803, p < 0.001) *and positively with γδTCR+ cells *(r = 0.603, p = 0.005*), CD16 correlates positively with γδTCR+ cells *(r = 1.138, p < 0.001*. There is no statistically significant correlation in old aged subjects.

**Figure 2:**
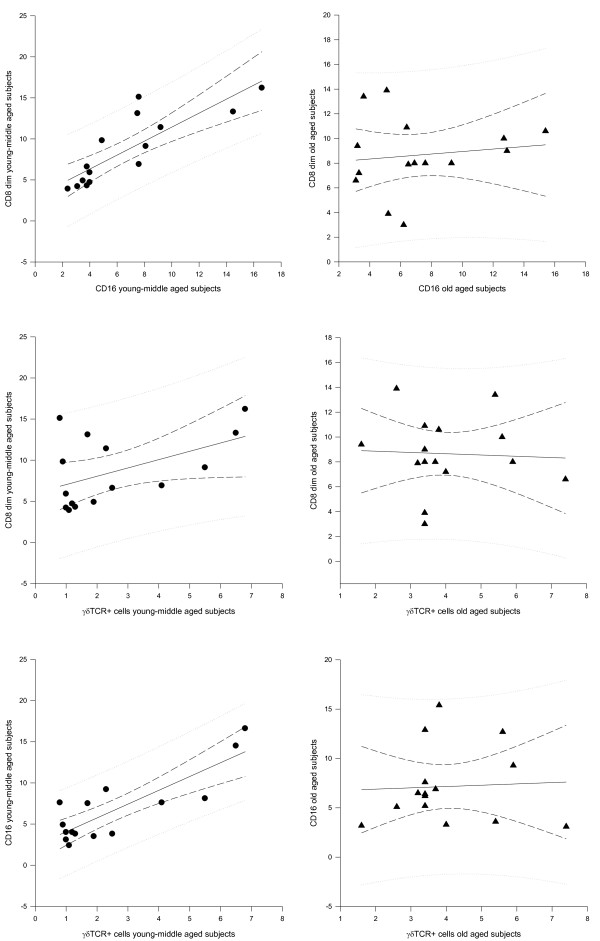
**Correlations between lymphocyte subpopulations in the scotoperiod**. In the scotoperiod (18:00h-22:00h-02:00h) CD8+ dim correlates positively with CD16+ *(r = 0.852, p < 0.001) *and positively with γδTCR+ cells *(r = 1.012, p = 0.05*), CD16+ correlates positively with γδTCR+ cells *(r = 1.676, p < 0.001)*. There is no statistically significant correlation in old aged subjects

Table 1 shows the human clusters of differentiations (CDs). Table 2 shows integrated time-qualified 24-hours values expressed as area under the curve (AUC) ± SE, with a statistically significant difference for the AUC values of CD20 (higher in young-middle aged subjects, p < 0.01) and for the AUC values of CD25, DR+ T cells and cortisol (higher in old aged subjects, p < 0.01, p = 0.01 and p = 0.04 respectively). Table 3 shows chronobiological data derived from best fitting sine curves (fitted period: 24 hours = 360°): in young middle aged subjects a clear circadian rhythm was validated for the time-qualified changes of CD3+, CD4+, CD20+, CD25+ and HLA-DR+ cells with acrophase during the night and for the time-qualified changes of CD8+, CD8+ bright, CD8+ dim, CD16+, γδTCR+ cells and cortisol with acrophase during the day. In old aged subjects a clear circadian rhythm was evidenced for the time-qualified changes of CD3+ and CD25+ cells with acrophase during the night and for the time-qualified changes of CD8+ cells with acrophase during the day. Figure 1 shows correlations among lymphocyte subpopulations in the photoperiod (06:00h-10:00h-14:00h): in young-middle aged subjects at 06:00 h CD8+ dim correlated positively with CD16+ (r = 0.803, p < 0.001) and positively with γδTCR+ cells (r = 0.603, p = 0.005), CD16 correlated positively with γδTCR+ cells (r = 1.138, p < 0.001), whereas in old aged subjects there was no statistically significant correlation among lymphocyte subpopulations. Figure 2 shows correlations among lymphocyte subpopulations in the scotoperiod (18:00h-22:00h-02:00h): in young-middle aged subjects at 18:00 h CD8+ dim correlated positively with CD16+ (r = 0.852, p < 0.001) and positively with γδTCR+ cells (r = 1.012, p = 0.05), CD16+ correlated positively with γδTCR+ cells (r = 1.676, p < 0.001), whereas in old aged subjects there was no statistically significant correlation among lymphocyte subpopulations. In young middle aged subjects cortisol correlated negatively with CD8+ dim (r = -0.472, p = 0.03) and with CD16 (r = -0.482, p = 0.01) at 18:00 h, whereas in old aged subjects cortisol correlated negatively with CD16 (r = -0.486, p = 0.04), with CD20 (r = -0.646, p < 0.001), with CD25 (r = -0.489, p = 0.04) and with γδTCR+ cells (r = -0.509, p = 0.02) at 06:00 h.

**Table 1:** Human Clusters of Differentiation (CDs)

CD3	the signaling component of the T cell receptor (TCR) complex, found on T cells
CD4	a co-receptor for MHC Class II, found on T helper/inducer subset
CD8	a co-receptor for MHC Class I, found on T suppressor/cytotoxic subset
CD16	FcγRIII, a low-affinity Fc receptor for IgG, found on NK cells, macrophages, and neutrophils
CD20	a type III transmembrane protein found on B cells
CD25	a type I transmembrane protein found on activated T cells that associates with CD122 to form a heterodimer that can act as a high-affinity receptor for IL-2
HLA-DR	a transmembrane human major histocompatibility complex (MHC) II family member expressed primarily on B cells on which it presents antigenic peptides for recognition by the T cell receptor on CD4+ T cells.
TcRδ1	epitope of the constant domain δ of chain of TCR found on γδTCR expressing cells

**Table 2:** Integrated time-qualified 24-hours values expressed as AUC ± SE

	Healthy young-middle aged subjects	Healthy old aged subjects
CD3	1545.41 ± 42.23	1576.07 ± 25.85
CD4	891.33 ± 60.52	837.41 ± 32.41
CD8	603.73 ± 92.12	615.24 ± 30.21
CD4/CD8 ratio	39.40 ± 12.71	31.53 ± 1.35
CD16	142.50 ± 45.30	171.72 ± 31.63
CD20	264.12 ± 30.84	132.78 ± 21.23^•^
CD25	76.12 ± 14.21	140.02 ± 24.25^•^
DR+T cells	61.8 ± 10.23	109.5 ± 8.31^•^
HLA-DR	327.05 ± 23.40	282.57 ± 20.42
TcRδ1	61.72 ± 13.71	86.23 ± 9.25
Cortisol	258.2 ± 13.4	310.6 ± 32.7^•^

Units: % for lymphocyte subpopulations, μg/dl for cortisol all; parameters analyzed in all the subjects; p < 0.05^•^

**Table 3:** Chronobiological data derived from best fitting sine curves (fitted period:24 hours = 360°)

Healthy young-middle aged subjects					
Factor	***p***	MESOR ± SE	Amplitude ± SE	Acrophase ± SE(°)	Time (Hh:Mn)
CD3	0.002	78.06 ± 0.10	1.12 ± 0.22	25.1 ± 12.4	01:40 ± 00:50
CD4	0.001	45.23 ± 0.85	3.14 ± 1.12	3.3 ± 24.5	00:13 ± 01:38
CD8	0.003	29.52 ± 0.23	1.94 ± 0.25	181.3 ± 2.4	12:05 ± 00:10
CD8 bright	0.001	21.43 ± 0.11	1.49 ± 0.21	187.3 ± 10.2	12:29 ± 00:41
CD8 dim	0.002	8.09 ± 0.14	1.33 ± 0.09	192.1 ± 3.8	12:48 ± 00:15
CD4/CD8 ratio	0.001	1.53 ± 0.02	0.23 ± 0.1	16.0 ± 0.2	01:04 ± 00:01
CD16	0.030	6.26 ± 0.42	0.81 ± 0.21	212.1 ± 21.3	14:08 ± 01:25
CD20	0.002	13.23 ± 0.24	1.51 ± 0.11	336.8 ± 12.2	22:27 ± 00:49
CD25	0.002	3.82 ± 0.02	0.67 ± 0.21	9.2 ± 7.1	00:37 ± 00:28
DR+T cells	0.005	3.21 ± 0.30	0.83 ± 0.20	12.2 ± 51.2	00:49 ± 03:25
HLA-DR	0.010	16.22 ± 0.25	1.33 ± 0.33	332.6 ± 11.3	22:10 ± 00:45
TcRδ1	0.002	2.12 ± 0.09	0.63 ± 0.12	159.5 ± 14.3	10:38 ± 00:57
Cortisol	0.001	10.23 ± 1.42	6.51 ± 2.52	141.6 ± 22.1	09:26 ± 01:28
					
**Old aged subjects**					
**Factor**	***p***	**MESOR ± SE**	**Amplitude ± SE**	**Acrophase ± SE (°)**	**Time (Hh:Mn)**
					
CD3	0.002	84.91 ± 0.21	1.07 ± 0.02	71.2 ± 3.0	04:45 ± 00:12
CD4	0.145	45.12 ± 0.89	3.10 ± 1.27	33.2 ± 22.1	02:13 ± 01:28
CD8	0.005	29.21 ± 0.23	3.25 ± 0.53	188.9 ± 11.2	12:36 ± 00:45
CD8 bright	0.001	20.12 ± 0.08	1.47 ± 0.19	196.1 ± 12.3	13:04 ± 00:49
CD8 dim	0.002	9.09 ± 0.15	1.31 ± 0.87	191.7 ± 2.4	12:47 ± 00:10
CD4/CD8 ratio	0.001	1.48 ± 0.06	0.22 ± 0.04	17.9 ± 11.7	01:12 ± 00:47
CD16	0.246	8.02 ± 0.31	2.42 ± 0.43	191.3 ± 7.5	12:45 ± 00:30
CD20	0.210	8.36 ± 0.16	1.15 ± 0.11	287.2 ± 21.4	19:09 ± 01:26
CD25	0.031	7.12 ± 0.13	1.02 ± 0.21	251.2 ± 12.3	16:45 ± 00:49
DR+T cells	0.057	5.12 ± 0.35	1.73 ± 0.2	141 ± 11.2	09:24 ± 00:45
HLA-DR	0.297	13.21 ± 0.12	1.21 ± 0.9	185.1 ± 33.2	12:20 ± 02:13
TcRδ1	0.210	4.28 ± 0.12	0.32 ± 0.11	192.1 ± 30.4	12:48 ± 02:02
Cortisol	0.017	13.26 ± 0.40	5.42 ± 1.31	121.8 ± 10.2	08:07 ± 00:40
					

Units: % for lymphocyte subpopulations, g/dl for cortisol, all parameters analyzed in all the subjects

**Figure 3:**
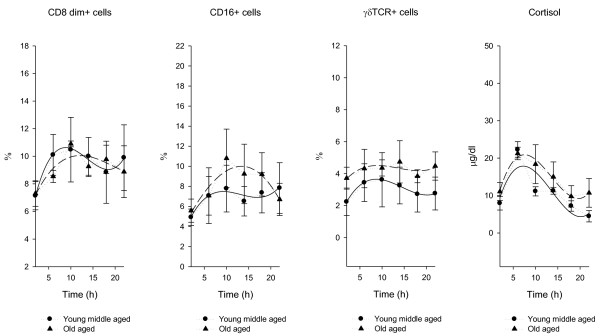
**X-Y plot showing 24-hour time qualified profiles of lymphocyte subset percentages and cortisol serum levels**. Values are expressed as mean ± SE calculated on single time point values from fifteen young-middle aged and fifteen old aged subjects. Percentages of circulating CD8 dim+, CD16+ and γδTCR+ T cell subpopulations and cortisol serum levels show circadian rhythmicity with acrophase during the day in young-middle aged subjects. In old aged subjects CD8 dim+ T cells and cortisol serum levels show circadian rhythmicity with acrophase during the day. Cubic regression function data interpolation represented as best fit solid line in young-middle aged subject and medium-dashed line in old aged subjects, superimposed on the raw data (dotted line).

Figure 3 shows 24-hour time qualified profiles of lymphocyte subset percentages and cortisol serum levels expressed as mean ± SE calculated on single time point values.

## Discussion

Cellular immune responses drive all adaptive immunity, lymphocyte subpopulations present circadian variation of some of their subsets and this variation may influence magnitude and expression of the immune responses [9-13]. Aging associated changes have been demonstrated not only in T lymphocytes but also in different aspects of the innate immunity including natural killer (NK) cells [14,15]. The CD8+ lymphocytes are heterogeneous in subphenotypes and functions and include T cells, which express high-density CD8 (CD4-CD8bright+) and T cells, which express low-density CD8 (CD4+CD8dim+). CD4+ T lymphocytes expressing CD8dim represent cytotoxic effector populations and contain high amounts of perforin, which explains their greater cytolytic capacity [16,17]. A distinct subset of CD3+CD4-CD8-T lymphocytes expresses a CD3-associated heterodimer made up of the protein encoded by the T-cell receptor (TCR) gamma-gene and a second glycoprotein termed TCR delta. TCR gamma-delta (γδ-TCR) is expressed on CD3+ thymocytes during fetal ontogeny before the appearance of TCR alpha-beta (γδ-TCR), on CD3+CD4-CD8-adult thymocytes, and on a subset (1-10%) of CD3+ cells in adult peripheral lymphoid organs and the peripheral blood. γδ-TCR expressing T cells probably represent a distinct mature T-cell lineage with the capacity to proliferate in response to receptor-mediated signals, and to display non-major histocompatibility complex (MHC)-restricted cytolysis [18,19]. NK cells are large granular lymphocytes that express neither α/β or γ/δ TCR nor CD3 on their surface, can lyse a number of different tumour cells and may be stimulated by IFN-γ, IL2, IL12 and IL18. The molecular and structural properties of γδ-TCR, the physiological role of T lymphocytes expressing γδ-TCR and the relationship between CD3+αβ T lymphocytes, NK and CD3+γδ T lymphocytes are still a matter of investigation.

There are different circadian variations of the total number of circulating immune cells and of specific lymphocyte subpopulations and the different compartmentalization of lymphocytes in space and in time has major functional consequences and leads to a partial fragmentation of immunoregulatory circuits at the local level [20-25]. The total number of circulating lymphocytes changes with circadian rhythmicity in antiphase with cortisol [26-28] and in our study we have evidenced that this rhythm of variation is recognizable for the changes of CD3 (total T cells), CD4 (T helper/inducer subset), CD20 (total B cells), CD25 (activated T lymphocytes with expression of α chain of IL2 receptor), HLA-DR (B cells and activated T cells) higher during the night, whereas CD8, CD8 bright and CD8 dim (T suppressor and cytotoxic lymphocytes respectively), CD16 (natural killer) and TcRδ1 (γδTCR expressing cells) are higher around noon. These opposing circadian variations resemble a temporal organization of cellular immune function. Naive T lymphocytes need to be activated and subsequently differentiate into effector cells to perform their immune functions. Regulation of T-cell responses involves diverse strategies of activation and inhibition to optimize recognition of infected or transformed cells, while preventing tissue damage as a result of autoreactivity and chronic inflammation. TCR-CD3-dependent responses are regulated by constitutive or inducible expression of costimulatory and inhibitory receptors, such as CD28 and its CTLA-4 counterpart. In recent years, however, it has become evident that the expression of NK cell receptors of the NKG2 family (eg, NKG2D and CD94/NKG2 receptors) on CD8+αβ+ effector T cells may represent another mean to regulate cytolytic functions in the tissue microenvironment, effectively controlling antigen-specific killing. NKG2D is one of the most widely distributed "NK-cell receptors," being expressed at the surface of all CD8+αβ+ T cells, γδ T cells, NK cells, as well as on certain activated CD4+ T cells. NKG2D is a potent costimulator of TCR-mediated effector functions and up-regulates antigen-specific T cell-mediated cytotoxicity directed against cells or tissues expressing stress-induced NKG2D ligands (NKG2DLs), particularly under conditions of suboptimal TCR engagement [29-36]. As evidenced in our study in healthy young-middle aged subjects the CD8dim+ T cytotoxic lymphocytes, NK cells and the γδ-TCR expressing cells show evident positive statistical correlation and a clear circadian rhythmicity of variation with higher levels during the photoperiod (figure 1,2 and 3) and as evidenced in the scientific literature they share costimulators and ligands, suggesting that cytotoxic cell compartment is tightly connected in time and maybe function. The surface molecules and the mechanisms involved in the activation of γδ-TCR+ cells are similar to those of αβ-TCR+ cells and activated γδ-TCR+ cells have strong cytotoxic effector activity using both death receptor/death ligand and cytolitic granule pathways and produce various cytokines, frequently including tumor necrosis factor-α and IFN-γ [37,38]. Most CD3+γδ expressing T cell lines mediate cytotoxicity against a broad spectrum of tumor-cell targets, although the functional significance of this observation remains unclear [39]. An hypothesis is that γδ-TCR expressing cells recognize subtle alterations in host cells that may be associated with neoplastic transformation. In our old aged subjects we have evidenced decrease of B cell compartment, that may cause diminished response to immunological stimulation, increase of activated T cell compartment, that may be responsible of increased autoimmunity phenomena and a severe alteration of circadian rhythmicity of variation of natural killer and γδ-TCR bearing cells with loss of physiological timed windows of interaction. This phenomenon may be very important and dangerous, considered that these cells might represent the true immune surveillance cells [40] and may contribute to the increased incidence of cancer in old aged people, working with the accumulating DNA damage produced by chemicals, physical agents, free radicals and a number of carcinogens widely contaminating the environment of our daily living.

In our old aged subjects we have evidenced higher cortisol serum levels with circadian rhythmicity of secretion characterized by advance in acrophase. These data are in agreement with those reported in the international literature describing that the circadian profile of plasma cortisol is conserved in the elderly, but with higher plasma levels during the night [41]. The human adrenals show a marked circadian periodicity in the response to endogenous ACTH, with an acrophase in the morning in young adult subjects and with relative resistance to endogenous ACTH stimulation in the evening hours. In the elderly this rhythm shows a marked decrease in amplitude, with similar response to ACTH during daytime and evening hours and this phenomenon causes an elevated cortisol 24 h mean level and a reduction in the rhythm amplitude[42]. The higher plasma cortisol levels at night may play a role in the cognitive and metabolic disturbances found in the elderly and in the immune changes found in our old aged subjects. Cortisol circadian rhythm is a robust rhythm that does not respond rapidly to minor and transient environmental changes, which makes it a good candidate as a rhythm marker, but a trend for a phase advance in plasma cortisol has been reported in the elderly [43]. The immune system function is characterized by a complex time structure composed of multiple rhythms in different frequency ranges. The rhythms of the same frequency may have the same phase or different phases and usually show a well defined time-relation to each other. The loss of the array of rhythms or a change of their functional interactions may alter the organism's time structure leading to chronodisruption and internal desynchronization. The alteration of the organism's time structure may lead to functional disturbances and may impair repairing and defensive mechanisms. A complete loss of rhythmicity or a change of phase of the rhythms are the most frequent alterations, but another important factor is represented by the change of amplitude of variation. The multifrequency structrure that characterizes the function of the immune system and the complexity of the time qualified variations of its different components has to be taken in consideration when we approach functional evaluations, clinical interpretations, and therapeutical interventions.

## Conclusion

The aging immune cellular system is characterized by a severe alteration of circadian rhythmicity of the cytotoxic compartment that may be responsible for functional derangement with increased susceptibility to and reduced defense against neoplastic disease.

## Competing interests

The authors declare that they have no competing interests.

In the past five years we have not received reimbursements, fees, funding, or salary from an organization that may in any way gain or lose financially from the publication of this manuscript, either now or in the future. No organization is financing this manuscript (including the article-processing charge).

We do not hold any stocks or shares in an organization that may in any way gain or lose financially from the publication of this manuscript, either now or in the future

We do not hold or are currently applying for any patents relating to the content of the manuscript. We have not received reimbursements, fees, funding, or salary from an organization that holds or has applied for patents relating to the content of the manuscript.

We have no other financial competing interests. There are no non-financial competing interests (political, personal, religious, ideological, academic, intellectual, commercial or any other) to declare in relation to this manuscript.

## Authors' contributions

GM: conception and design of the study, data collection, analysis and interpretation of data, carried out statistical analysis and the draft of the manuscript.

AD: interpretation of data, carried out part of the draft of the manuscript.

AG: interpretation of data, carried out part of the draft of the manuscript.

MD: data collection, data interpretation, carried out part of the draft of the manuscript

NM: data collection, data interpretation,

MPD: data collection, data interpretation, carried out part of the draft of the manuscript

SC: data collection, data interpretation,

FP: data interpretation, carried out part of the draft of the manuscript

RT: critical revisal of the manuscript, interpretation of data

All the Authors have read and approved the submission of the present version of the manuscript and that the manuscript has not published and is not being considered for publication elsewhere in whole or in part in any language except as an abstract.
